# Center volume, competition, and outcome in German liver transplant centers

**DOI:** 10.1186/2047-1440-3-6

**Published:** 2014-02-10

**Authors:** Markus Guba

**Affiliations:** 1 Department of General, Visceral, Transplantation, Vascular and Thoracic Surgery, University of Munich, Transplant Center Munich, Munich, Germany

## 

In recent years, a growing body of literature has suggested that patients who undergo surgery at high-volume centers fare better than those treated in low-volume hospitals. In the study by Nijboer et al. (unpublished work) in this month’s *Transplant Research* issue, liver transplantation outcomes were investigated in relation to center case volume in 24 German transplant centers. High-volume centers had higher 1-year survival rates, but no difference in in-hospital mortality. The overall outcomes of all German transplant centers were poor with in hospital mortality rate of 18% and a 1-year patient survival of 73%. The ROC analysis did not reveal a clinical meaningful cutoff value for low- *versus* high-volume center. Looking at the center data in Nijboer’s study, one gets the impression that the volume outcome relationship is not linear but a bell-shaped curve with inferior outcomes in small centers (<20) and centers with very high volume (>80-100). Centers with a volume below 20 cases per year show very heterogeneous results, suggesting that some centers in that group may not have the personal and infrastructural resources to run the program. On the other hand, very high-volume centers may be pressured to keep their high market share by accepting marginal transplant candidates and a poor donor organ/recipient match. A recently published article by Macomber et al. has analyzed for the same purpose the UHC database which included 63 US liver transplant centers [1]. What they found was that high-volume centers, defined as those performing more than 75 liver transplants per year had lower morbidity and mortality rates than lower-volume centers and were also more cost-efficient. To provide easy adequate access for patients in some German regions the establishment of small-volume centers may be necessary, but in those where such issues are not so pressing it may become difficult to justify having higher-cost and worse-performing small centers, as a high-performing lower-cost center. In Germany such decisions usually lie in the hands of the federal states, but in close-by centers patients may well vote with their feet if the data are made public. Besides volume another important factor, hospital competition, may negatively interfere with outcome. Although more hospital competition is associated with improved access to care, it also can be linked to poorer outcomes, according to a recent study by Halldorson et al. [2]. The authors found that low, mid, and high levels of competition significantly increased the risk of graft failure and patient death. With increased competition among medical centers that perform liver transplants, higher-risk patients received lower-quality donor organs. Transplantation for sicker patients and the use of higher-risk organs also meant higher costs. The findings question whether competition for the same organs decreased the ability to match donor and recipient characteristics. Competition may incentivize medical centers to perform enough transplants to meet fixed costs, boost profits, and maintain market share, which can inhibit them from best matching organs with recipients. Competition has been advocated as important for bettering market performance, but there may be limits to the value of competition in the healthcare setting. The debate about volume, competition, and transparent outcome reporting continues. Liver transplantation is often looked on as a ‘prestige programme’ and healthcare providers are reluctant to give up their (small and not cost-effective) liver transplant program. In the light of unacceptable poor outcomes in liver transplantation in Germany, the study by Nijboer et al. will contribute to an ongoing discussion on the size and organization of transplant centers.
